# High quality copy number and genotype data from FFPE samples using Molecular Inversion Probe (MIP) microarrays

**DOI:** 10.1186/1755-8794-2-8

**Published:** 2009-02-19

**Authors:** Yuker Wang, Victoria EH Carlton, George Karlin-Neumann, Ronald Sapolsky, Li Zhang, Martin Moorhead, Zhigang C Wang, Andrea L Richardson, Robert Warren, Axel Walther, Melissa Bondy, Aysegul Sahin, Ralf Krahe, Musaffe Tuna, Patricia A Thompson, Paul T Spellman, Joe W Gray, Gordon B Mills, Malek Faham

**Affiliations:** 1 Affymetrix Inc. Santa Clara, CA, USA; 2 MD Anderson Cancer Center, Houston, TX, USA; 3 DF/BWH Cancer Center, Boston, MA, USA; 4 University of California San Francisco, San Francisco, CA, USA; 5 Cancer Research UK, London Research Institute, London, UK; 6 Arizona Cancer Center, Tucson, AZ, USA; 7 Lawrence Berkeley National Laboratory, Berkeley, CA, USA

## Abstract

**Background:**

A major challenge facing DNA copy number (CN) studies of tumors is that most banked samples with extensive clinical follow-up information are Formalin-Fixed Paraffin Embedded (FFPE). DNA from FFPE samples generally underperforms or suffers high failure rates compared to fresh frozen samples because of DNA degradation and cross-linking during FFPE fixation and processing. As FFPE protocols may vary widely between labs and samples may be stored for decades at room temperature, an ideal FFPE CN technology should work on diverse sample sets. Molecular Inversion Probe (MIP) technology has been applied successfully to obtain high quality CN and genotype data from cell line and frozen tumor DNA. Since the MIP probes require only a small (~40 bp) target binding site, we reasoned they may be well suited to assess degraded FFPE DNA. We assessed CN with a MIP panel of 50,000 markers in 93 FFPE tumor samples from 7 diverse collections. For 38 FFPE samples from three collections we were also able to asses CN in matched fresh frozen tumor tissue.

**Results:**

Using an input of 37 ng genomic DNA, we generated high quality CN data with MIP technology in 88% of FFPE samples from seven diverse collections. When matched fresh frozen tissue was available, the performance of FFPE DNA was comparable to that of DNA obtained from matched frozen tumor (genotype concordance averaged 99.9%), with only a modest loss in performance in FFPE.

**Conclusion:**

MIP technology can be used to generate high quality CN and genotype data in FFPE as well as fresh frozen samples.

## Background

DNA copy number (CN) studies hold great promise for the discovery of clinical biomarkers to predict disease course, recurrence risk, and response to therapy. A recent meta-analysis has confirmed that the efficacy of highly toxic anthracyclines in early breast cancer is limited to women with HER2 (ERBB2) amplification or overexpression [1] and EGFR copy number holds promise in predicting response to the expensive monoclonal cetuximab, in contrast to the apparent failure of immunohistochemical staining for EFGR [2]. While exciting results have been found with genes already known to be involved in key pathways, confirming early results and genome-wide testing requires large numbers of well-characterized clinical samples. A vast collection of stored FFPE samples already exists; extrapolation from the Genome Austrian Tissue Bank numbers suggests that ~500 million FFPE samples may have been collected in North America and Europe alone during the last quarter century [3]. Unfortunately many genomic assays fail to produce high quality CN and genotype data from FFPE samples [4-10], restricting the application of these promising whole genome scanning technologies to the limited number of fresh frozen samples.

The FFPE process was developed over a hundred years ago, long before pathologists were concerned with the preservation of DNA. Sample DNA is often damaged by exposure to formaldehyde and a potentially extremely acidic environment [11,12]. This degradation creates short DNA fragments that are potentially unsuitable for existing high density CN platforms. In addition, the chemical damage and modifications that FFPE DNA may suffer from can inhibit the enzyme-dependent chemistries necessary for a number of approaches [11]. This damage is reflected in the high rate of sequencing artifacts and genotyping failures seen with FFPE extracted DNA [13,14]. Finally, often only limited tumor DNA is available from FFPE samples (particularly those from needle biopsies or small early stage tumors) while some CN platforms require large amounts of DNA. Few studies have compared CN results in matched fresh frozen and FFPE samples and these studies have been small (with generally less than 20 fresh frozen/FFPE pairs). Some have reported spurious copy number changes in FFPE samples and generally FFPE samples fail more often than fresh frozen. When genotypes are also measured, FFPE samples have substantially lower call rates (suggesting a loss of performance) and genotype discordances between FFPE and fresh frozen samples raise troubling questions about data reliability [5-10].

Molecular Inversion Probe (MIP) technology offers a potential solution to the challenges of CN and genotype assessment in FFPE-derived DNA samples. The small intact target DNA sequence footprint required by MIP probes (~40 bp) makes the MIP platform well suited to working with degraded FFPE DNA. MIP has previously been used to obtain high quality CN and genotype data from cell lines and frozen tissues and requires less than 100 ng of input DNA [15]. In this study, we show the successful application of the MIP technology in obtaining high quality CN and genotype data from seven diverse sets of FFPE samples (Table 1).

**Table 1:** FFPE Samples used in the study

**Institution**	**Tumor sample type**	**Age range of Blocks (years)**	**Number of FFPE tumors**	**Number of FFPE normals**	**Other features**
MD Anderson set 1	Breast cancer	< 3	8	9	

MD Anderson set 2	Breast cancer	5 – 22	27	18	

UCSF	Liver metastases from colorectal	5 – 28	9	9	Matching frozen tumors and normals available

Dana Farber	Invasive breast cancer	5 – 6	6	13	Microdissected

CRUK	Colorectal	0.5 – 5	17	16	Matching frozen tumors available; macrodissected

CHTN	Bladder, colorectal, kidney, liver	1 – 3	13	15	Matching frozen tumors and normals available

Leader	Kidney	3 – 4	13	12	

## Methods

### Samples

Informed consent was obtained from all subjects and study protocols approved by the relevant institutional review boards. Sample information is provided in table 1. The Dana Farber tumor samples underwent manual microdissection to remove stromal components from H&E stained FFPE sections. Other sites/sources of samples were: the Cooperative Human Tissue Network (CHTN); Cancer Research UK (CRUK); Leader, Inc. ; MD Anderson Cancer Center; and University of California, San Francisco (UCSF). For the UCSF samples only, CN data generated with a different technology (BAC arrays) were available.

### MIP assay and analysis

The basic MIP assay has been previously described [16-19]. MIP probes are oligonucleotides in which the two end sequences are complementary to two adjacent genomic sequences; these two ends anneal to the genomic DNA in an inverted fashion with a single base between them (generally the site of a single nucleotide polymorphism; SNP). In CN analysis, genomic DNA is hybridized to the MIP probe and the reaction split into two separate tubes containing paired nucleotide mixes (triphosphates of either Adenine + Thymine or Cytosine + Guanine) [15]. With the addition of polymerase and ligase, the MIP probe circularizes in the presence of the nucleotide complementary to the allele on the genome. For the assessment of FFPE samples, we used 4X the amount of ligase and polymerase as compared to the traditional protocol. Genomic DNA is limiting in the reaction such that the number of circularized probes proportionally reflects the absolute amount of template DNA. After circularization, unused probes and genomic DNA are efficiently removed from the reaction by exonuclease leaving only circularized probes. These probes are then amplified, labeled, detected, and quantified by hybridization to tag microarrays; tags are designed to have low cross hybridization. An important advantage of the MIP technology is the allele discrimination is performed enzymatically and is highly specific, allowing highly multiplexed assays (> 50,000 markers) with very precise quantitation of signals. The probes used in this panel are listed in Additional file 1.

The MIP assay and CN determination have been described previously [15]. Since the amount of tumor DNA is often limiting we have implemented a change in the assay to use 37 ng of DNA, half the amount that was used previously (75 ng). This input amount was selected after a set of experiments using various starting amounts of DNA (2-fold steps) and probe concentrations. Dropping the input amount did not yield robust results with the current assay conditions (data not shown). The other change is that an optimal reference was chosen for each tumor. Reference selection was guided by signals between tumor and reference samples; specifically we summed signal for the two alleles of each marker and measured between sample sum_signal correlations. We found that selecting a reference set with high correlation led to better CN data in the tumor. Often we found that the normal samples from a study site had the highest correlation with tumor samples and hence just used site-specific normals. If the site-specific normals did not show the highest correlation (as was the case about half of the time), we selected the 3–10 normals with the highest correlation as the reference set (the number of normals picked was based on correlation; if 5 normals had high correlation with the tumor and 6^th ^normal had much lower correlation, we used just 5 normals).

ROC curves were generated for samples with known copy number changes; specifically we looked at copy number in normal male fresh frozen and FFPE samples. For X-chromosome markers we expect CN = 1, so if the CN was below the cut-off threshold and we inferred CN = 1, we called it a true positive; if it was above and we inferred CN = 2, we called it a false negative. For autosomal markers we conservatively assumed all markers should have CN = 2 with no copy number variations, so if the CN was below the cut-off threshold and we inferred CN = 1, we called it a false positive; if it was above and we inferred CN = 2, we called it a true negative. The curve is generated by varying the cut-off threshold. In order to compare the 2p-RSE to the ROC curves, we needed to summarize the ROC curve data to a single value and chose the false positive rate at 50% sensitivity (FPR_50_). In order to study this comparison over a broad range, samples exhibiting a relative large range of quality (i.e. large range of 2p-RSE values) were sought. Since the reference set used is an important determinant of the quality of the CN data, we purposely used several reference sets for each sample to obtain a wide range of quality in the CN data.

## Results

### Metrics to evaluate CN performance: ROC and 2p-RSE

The algorithm for estimation of CN has been previously described [20]. Briefly, a set of normal samples (assumed copy number 2) is used as a reference to establish the relationship between signal and CN on a marker-by-marker basis, with each allele of a marker assessed independently. Signals from test samples can then be converted to CN estimates using the signal/CN relationship observed in the reference set. While in previous studies with fresh frozen and cell line DNA, we found that the reference set selection had little impact on final data quality, we noted that for FFPE samples reference set selection markedly affected data quality. Therefore to obtain "optimal" data quality we developed a simple algorithm to "pick" the best reference sets (see methods).

We have previously described the performance of the MIP technology using receiver operating characteristic (ROC) curves with true positive/false positive analysis in samples with known CN changes (such as males with one copy of the X-chromosome) [15]. As ROC curves are useful only for assessing results in samples with known CN changes, we have developed a per sample data quality metric for this study that could be assessed in tumor samples with unknown CN changes.

The two-point relative standard error (2p-RSE) measures the noise in CN data based on the fact that in almost all cases we expect two adjacent markers to have the same true CN; even in highly unstable tumors, the number of expected amplification/deletion breakpoints should be substantially less than the number of markers in our panel, hence the variation in CN between two adjacent markers is, in most cases, caused by noise. Hence, the median relative standard error value (2p-RSE) among all pairs of adjacent markers in a sample reflects experimental noise. The 2p-RSE is a per sample metric that can be assessed in samples with unknown CN changes.

**Figure 1:**
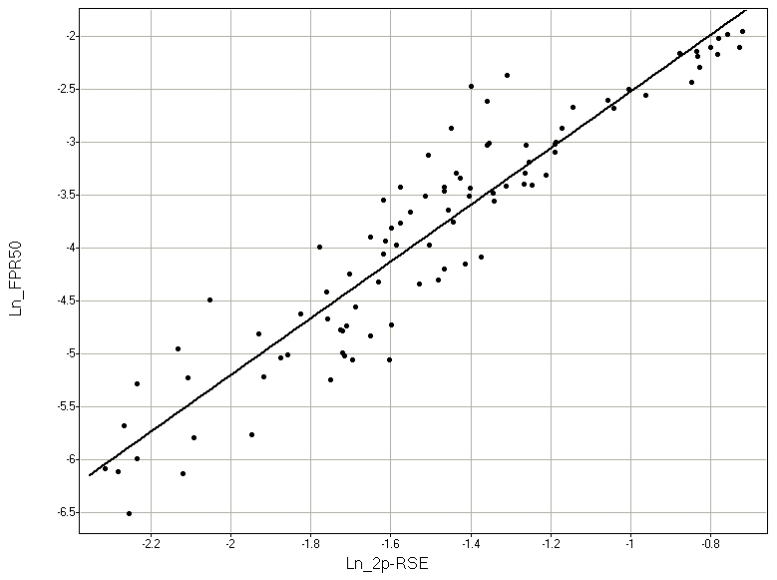
**Relationship between the natural log of the false positive rate at 50% sensitivity (Ln_FPR50) and the natural log of the 2p-RSE (Ln_2p-RSE)**. Each point represents a single sample with copy number assessed using a particular reference set. Samples were assessed with multiple reference sets and hence may appear multiple times in the figure. In samples for which we can asses both the false positive rate at 50% sensitivity (FPR50) and 2p-RSE, the two metrics show a close relationship.

To validate 2p-RSE as a sample metric, we looked at its correlation with ROC curves in samples with known CN. Each ROC curve was simplified to a single metric (the false positive rate at 50% sensitivity [FPR_50_], i.e. what percentage of positives is false when the sensitivity is sufficient to detect 50% of true positives?). The FPR_50 _and 2p-RSE were highly related; specifically their natural logs have a linear relationship [figure 1; ln(2p-RSE) = -0.25 + 0.32 * ln(FPR_50_)] with an r^2 ^of 0.87. Based on these data, we set a per sample passing threshold of 2p-RSE ≤ 0.25, which corresponds to a per marker (unsmoothed) FPR_50 _of approximately 3%. We also set a "high quality" sample threshold of 2p-RSE ≤ 0.18, which corresponds to a per marker FPR_50 _of approximately 1.5%. Of 93 FFPE tumor samples with sufficient DNA, 82 (88%) passed, and of these 62 (76%) met the high quality threshold (Table 2 and Additional file 2). All of the 39 fresh frozen tumor samples passed the high quality threshold.

**Table 2:** CN Performance of different FFPE and fresh frozen sets

**Institution (type)**	**# with sufficient DNA**	**# passed (% of all)**	**# high quality (% of passed)**	**median 2p-RSE of passed**
CHTN (FFPE)	13	13 (100%)	11 (85%)	0.139

CHTN (FF)	12	12 (100%)	12 (100%)	0.102

CRUK (FFPE)	17	16 (94%)	12 (75%)	0.163

CRUK (FF)	17	17 (100%)	17 (100%)	0.142

Dana Farber (FFPE)	6	6 (100%)	1 (17%)	0.201

Leader (FFPE)	13	7 (54%)	4 (57%)	0.155

MD Anderson set1 (FFPE)	8	8 (100%)	7 (88%)	0.163

MD Anderson set2 (FFPE)	27	23 (85%)	18 (78%)	0.167

UCSF (FFPE)	9	9 (100%)	9 (100%)	0.115

UCSF (FF)	10	10 (100%)	10 (100%)	0.116

**All FFPE**	93	82 (88%)	62 (76%)	0.16

All FF*	39	39 (100%)	39 (100%)	0.129

* Fresh frozen

**Figure 2:**
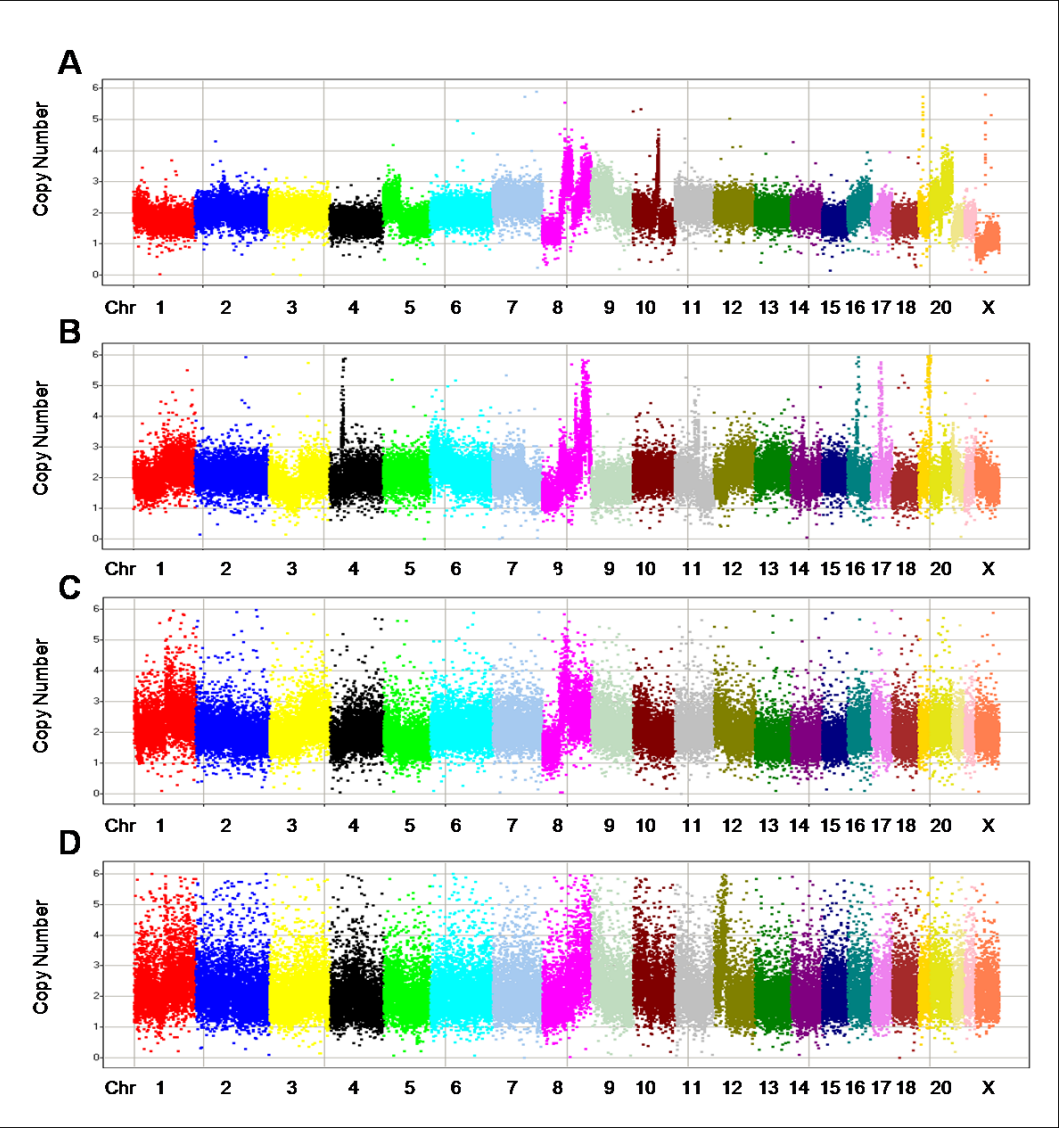
**Copy number data for 4 samples of varying 2p-RSEs**. In each panel, markers are arranged along the chromosomes and colored by chromosome. Chromosomes are typically labeled; some of the smaller chromosomes are unlabeled due to space constraints. The X axis represents chromosomes in genomic order of markers; the Y axis is the absolute copy number measurement. (A) shows one of the best samples with 2p-RSE = 0.109. (B) shows an average "high quality" sample with 2p-RSE = 0.147. (C) shows a sample that just fails to meet the high quality threshold with 2p-RSE = 0.184. (D) shows the worst passed sample with 2p-RSE = 0.247.

Figure 2 shows CN data for a cross-section of FFPE tumor samples with different 2p-RSEs: one of the best samples (A), a median "high quality" sample (B), a passed, non-"high quality" sample (C), and the worst passed sample (D).

### Evaluation of 7 FFPE sample collections

#### Genotyping

The MIP technology generates marker genotype information as well as CN. Genotypes can be useful in detecting sample tracking errors, assessing data quality, identifying copy-neutral loss-of-heterozygosity (LOH), and recognizing allelic bias in copy number changes. Genotyping of tumors is complicated by CN changes and normal tissue contamination, but to a first approximation, tumors can be genotyped as homozygous for either allele or heterozygous.

To detect sample tracking errors, we compared genotypes between all samples. Genotype concordance were either high (> 94%, presumably for samples derived from the same individual) or low (< 82%, presumably for samples derived from different individuals). Genotype concordances pointed to three sample tracking errors ("matched" sample with concordances below 82%) and in all three cases we could determine which sample was mistracked.

Ability to genotype and genotype concordance both reflect data quality. To pass genotyping, we required that a sample have a call rate above 85% although most passed samples had far higher call rates (average 98.5%, median 99.4%). Of 168 FFPE samples (93 tumors and 75 normals), 96.4% passed genotyping and all 61 fresh frozen samples (39 tumors and 22 normals) passed genotyping. We found that the average call rate was only slightly lower in FFPE tumors than fresh frozen tumors (97.5% versus 98.7%). Consistent with previous reports, we found higher call rates in normal samples (98.9% FFPE and 99.8% fresh frozen) than tumors [6]. Genotype call rate was inversely correlated with 2p-RSE (r^2 ^= 0.54).

Two normal samples from the same individual should have exactly the same genotypes and differences indicate data errors. When we compared 20 normal pairs (FFPE versus fresh frozen), the average genotype concordance was 99.99% (range 99.93–100%). We expect slightly lower concordance in comparisons between tumor FFPE and fresh frozen samples as these samples are drawn from different portions of a tumor and genetic heterogeneity may lead to real differences; in 37 tumor pairs the average concordance was 99.90% (range 99.48–99.99%).

Simple genotype concordance between normal and tumor tissues can be depressed by CN changes in the tumor. Hence we instead assessed incompatible genotypes in the tumor; if the normal tissue is homozygous for allele A, any tumor genotype that has allele B (AB or BB) is incompatible with the normal genotype. (Theoretically rare somatic mutations at the site of a SNP could create incompatibility, but we conservatively assumed that all incompatibilities were the result of genotyping errors.) Comparison of genotypes in matched tumor and normal pairs found high compatibility; compatibility in 89 pairs was above 98.43%, with the exception of one sample that had 96.85% compatibility. CN analysis indicated that in this case the tumor and normal sample had been switched ("tumor" was all CN = 2, while "normal" had multiple CN changes). Correcting for this sample switch raised compatibility to 99.85%. Further CN analysis of samples indicated another sample tracking error in which both "normal" and "tumor" samples appeared to be derived from tumor tissue. We found 5 sample tracking errors in 229 samples; sample tracking errors are expected when large numbers of samples are manually handled [21,22].

As can be seen in Table 3, the percentage of incompatible genotypes in the 88 passed, matching normal-tumor pairs (after excluding the pairs mentioned above) was low in all the collections, but tended to be higher in FFPE pairs than fresh frozen pairs (average 0.1% versus 0.01%). This difference appears to be largely due to FFPE samples with low genotyping call rates, which tended to correlate with increased genotyping inconsistencies (Additional file 2).

**Table 3:** Genotype Performance of different passed FFPE and fresh frozen sample sets

**Institution (type)**	**Median call rate (%)**	**# pairs^**	**Median rate genotype inconsistencies**	**Range genotype inconsistencies**
CHTN (FF)	99.6	12	7.10E-05	2E-5 – 6E-4

CHTN (FFPE)	98.6	11	1.60E-04	2E-5 – 3E-3

CRUK (FFPE)	99.2	14	1.10E-04	2E-5 – 2E-4

Dana Farber (FFPE)	93.9	6	2.80E-03	3E-4 – 8E-3

Leader (FFPE)	98.5	9	2.40E-04	6E-5 – 1E-3

MD Anderson set1 (FFPE)	97.5	6	4.90E-04	6E-5 – 9E-4

MD Anderson set2 (FFPE)	97.8	13	2.70E-04	4E-5 – 2E-2

UCSF (FF)	98.9	10	2.00E-05	2E-5 – 2E-4

UCSF (FFPE)	99.2	7	7.10E-05	2E-5 – 1E-4

**All FFPE**	98.4	66	2.70E-04	2E-5 – 2E-2

All FF*	99.4	22	4.00E-05	2E-5 – 6E-4

^ The number of pairs with available data allowing the Mendelian inconsistency calculation

* Fresh frozen

#### Allelic ratios

**Figure 3:**
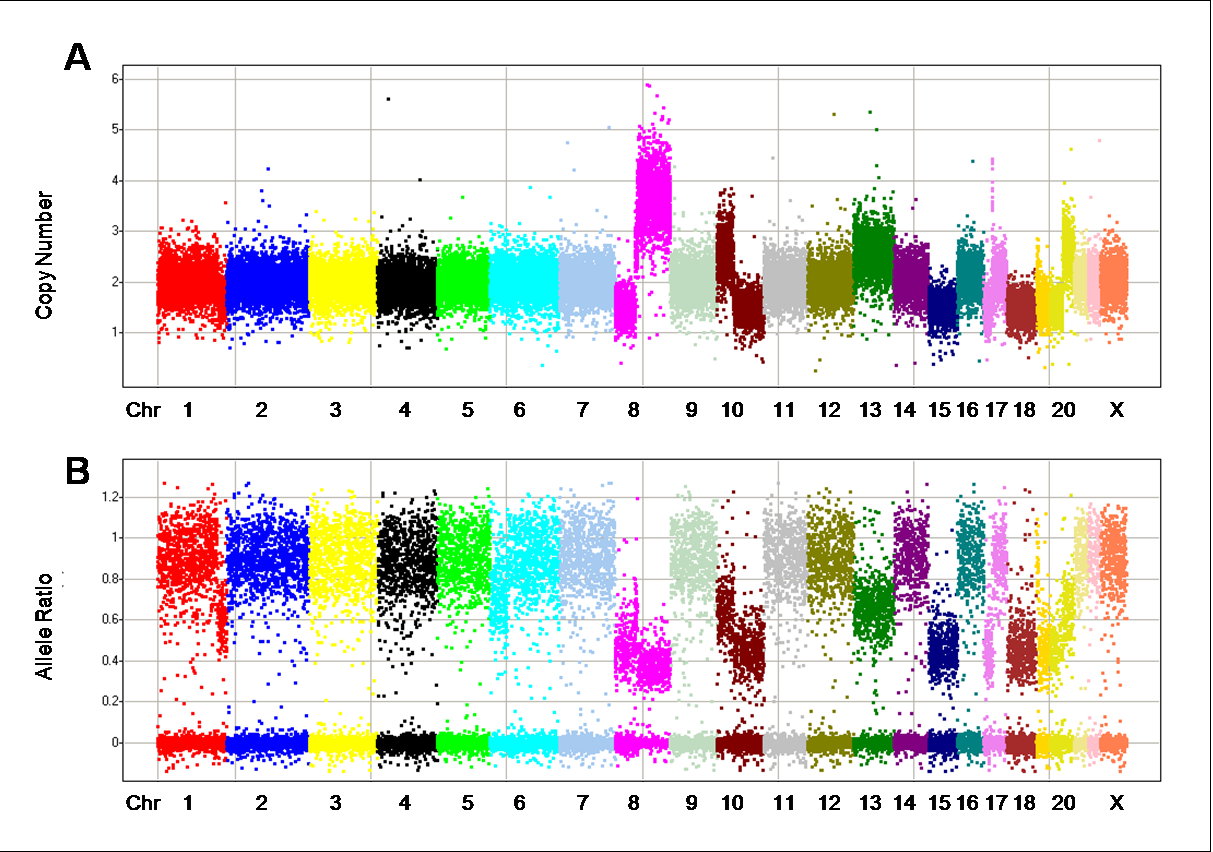
**Copy number (A) and allele ratio (B) data from the same tumor illustrating how the information can be used in concert to infer stromal contamination**. In each panel, markers are arranged along the chromosomes and colored by chromosome. Chromosomes are typically labeled; some of the smaller chromosomes are unlabeled due to space constraints.

Separately measuring copy number for each allele allows us to determine the allele ratio (AR); AR = CN_allele1/CN_allele2, where allele2 is the allele with the larger CN (a caveat described in Wang at al. [15] causes the allele ratio to occasionally be greater than 1). In a simple diploid sample with no copy number changes, the allele ratio should either be 0 (homozygous) or near 1 (heterozygous). The allele ratio can be helpful in identifying LOH events (including CN-neutral events), and through these estimating the level of stromal tissue contamination in a sample. LOH events in the absence of stromal contamination should mimic homozygosity. Figures 3A and 3B show the CN and AR for a sample with multiple CN changes. In figure 3B homozygous markers have an allele ratio that clusters very tightly around zero, but there are no regions in which all markers appear to be homozygous with AR = 0 (i.e. there are no regions that show complete LOH).

Most biopsy samples contain normal, stromal tissue and in the presence of this type of contamination we do not expect to find regions of complete LOH; if malignant cells in the tumor have undergone a deletion, the presence of stromal contamination will generate ARs above 0 but below 1. Formulas for determining the relationship between the true and apparent CN and AR given y% contamination with normal DNA are shown in Additional file 3. The data suggest that ~45% of the "usable" DNA in the figure 3 sample comes from CN = 2 cells, the majority of which are likely to be normal cells. Assuming such a level of contamination, we would expect copy loss LOH events (e.g., chromosomes 15 and 18, and parts of chromosomes 8, 10, 17, 19 and 20) to have a CN value around 1.45 and AR values around 0.45, which are close to the values seen. For amplification events where the tumor has 2 copies of one chromosome and 1 copy of the other (e.g., chromosomes 13 and part of chromosomes 20), we expect CN values to be around 2.55 and AR values around 0.65, again similar to the values seen. Finally, for amplification events where the tumor has four copies of one chromosome and one copy of the other (e.g., the qter of chromosome 8), we expect a CN value around 3.65 and AR value around 0.38. Chromosome 8 (in bright pink) is particularly interesting in that it appears to have a true CN of 1 from pter to ~34 Mb, then a region of CN = 3 from ~34 Mb to ~43 Mb, and finally a region of CN = 5 (where one copy of the chromosome was amplified to four copies) from ~43 Mb to qter. Inferring true CN may be even more complex if the tumor shows increased ploidy or genetic heterogeneity.

#### CN assessment

Of 93 FFPE tumor samples with sufficient DNA, 82 (88%) passed our 2p-RSE threshold, and of these 62 (76%) met the high quality threshold (Table 2). (As normal samples are typically used as references, meaningful 2p-RSEs were not measured in these samples.) For three of the collections, fresh frozen samples from the same individuals were also available for testing. For the UCSF collection, the median 2p-RSE for the fresh frozen and the FFPE tumor samples was essentially identical and for the CHTN and CRUK collections, the FFPE performance as measured by the median 2p-RSE of the FFPE samples was slightly worse than that of the fresh frozen samples (Table 2 and Additional file 2).

**Figure 4:**
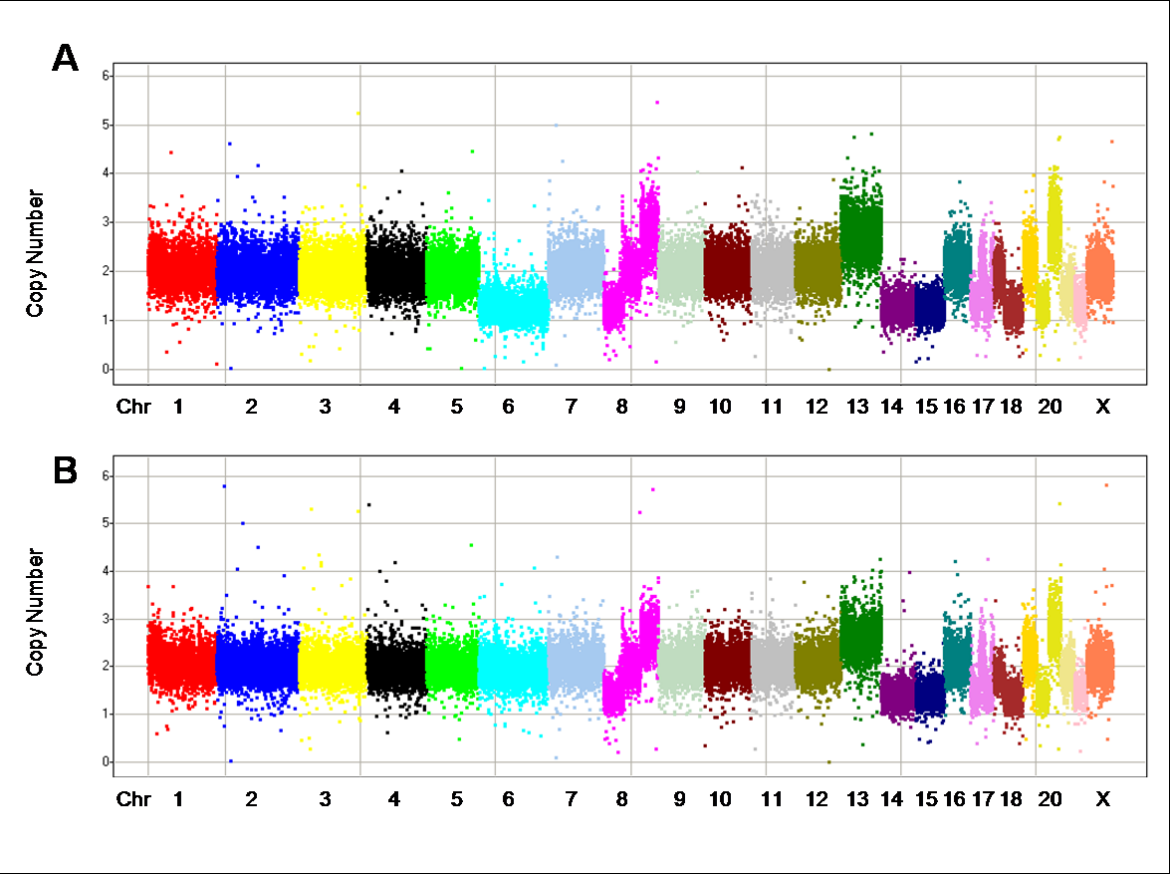
**CN in FFPE (A) and fresh frozen (B) samples from the same tumor**. In each panel, markers are arranged along the chromosomes and colored by chromosome. Chromosomes are typically labeled; some of the smaller chromosomes are unlabeled due to space constraints.

CN profiles for FFPE and frozen tissue from the same tumor were generally very similar: see example in figures 4A and 4B (full CN and AR results are provided in Additional files 4 and 5 and in GEO [; GSE14353, GSE14740–GSE14745]; a translation table for probes is in Additional file 1 and for samples in Additional file 2). For some tumors, there were differences in CN between samples, suggestive of genetic heterogeneity. Figure 4 shows one CN change between an FFPE tumor and a matched fresh frozen sample. The FFPE sample (A) shows a CN reduction for chromosome 6, whereas the fresh frozen sample (B) appears to be near CN = 2. Detailed examination found that in the fresh frozen sample the average CN for chromosome 6 is only 1.9 rather than 2, and the allele ratio (data not shown) indicates a partial loss-of-heterozygosity in the frozen sample. We suspect that differences are due to tumor heterogeneity: the FFPE sample has undergone a complete loss of one copy of chromosome 6 and the frozen sample is a heterogeneous mix of cells with and without the loss.

When we further evaluated differences between two samples from the same tumor, the samples appeared to share a similar underlying pattern of CN changes with one of the samples sometimes harboring additional changes (*i.e.*, one of the two samples appeared to match a presumed "ancestral" state as is seen for the chromosome 6 changes in the sample in figure 4) (data not shown).

**Figure 5:**
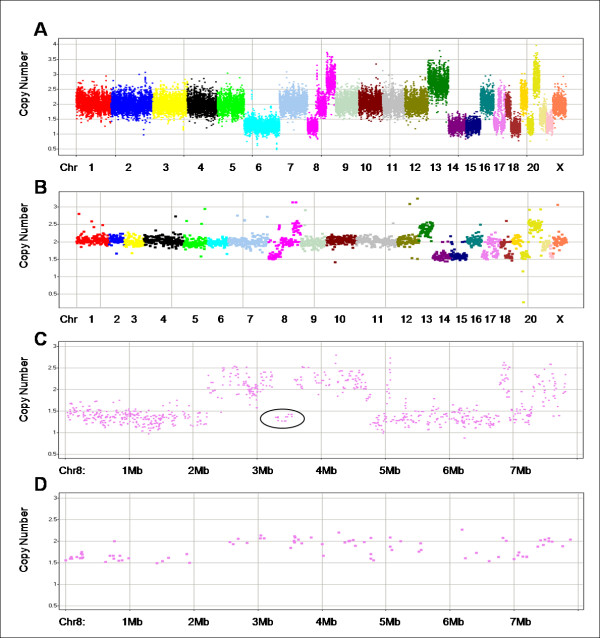
**Comparison of MIP and BAC CN results from the same tumor as shown in figure 4**. Markers are arranged along the chromosomes and colored by chromosome. Chromosomes are typically labeled; some of the smaller chromosomes are unlabeled due to space constraints. (A) shows the MIP data for the FFPE sample in figure 4B after smoothing (simple 3 marker median) and (B) shows the BAC data. (The two panels are on different X-axis scales because of differences in marker densities. However the panels use the same chromosome color coding scheme to facilitate comparisons. Differences in chromosome 6 are like those seen in figure 4 and, as we discuss in the text, likely due to tumor heterogeneity.) The next two panels show chromosome 17 MIP (C) and BAC (D) data. (These two panels share the same scale on the x-axis.) Some of the fine structure seen in the MIP data is missing in the BAC data, potentially due to resolution differences or genetic heterogeneity: an example is circled.

Eight of the fresh frozen samples used in this study were previously tested with BAC arrays [23]. The large segments of gain/loss from the BAC data were similar to those obtained with MIP from the corresponding FFPE samples [an example is shown in figures 5A (MIP) and 5B (BAC)]. (This sample is also shown in figure 4 and, as discussed previously, the tumor appears to be genetically heterogeneous for chromosome 6. The region of the tumor used to generate the FFPE block and then tested with MIP has CN = 1 for chromosome 6. The region of the tumor that was fresh frozen has a copy number near 2; using both BAC and MIP chromosome 6 has a slightly lower copy number than other apparent CN = 2 regions, suggesting a mixture of CN = 1 and CN = 2 cells, with CN = 2 cells predominating.) Some of the finer aberrations found by MIP were not seen by the lower resolution BACs [figures 5C (MIP) and 5D (BAC)].

We also briefly looked at CN patterns to identify common amplifications or deletions in these tumors. Because our samples are from such diverse types of tumors (the study was designed to test reliability of the technology with diverse samples), we lack the power to systematically identify CN patterns. We note, however, that the most common amplifications gains of 8q and 20q (both seen in ~30 FFPE samples) and the most common loss was of 8p, seen in 15 FFPE samples. These three changes are all commonly reported in tumors [24-26].

## Discussion

We previously demonstrated the ability of MIP technology to generate high quality allelic CN data from cell lines and frozen samples [15]. We have now applied this technology to archival tumor material and developed an analytical framework to obtain high quality CN and genotype data for DNA extracted from FFPE samples. We have developed a new data quality metric, the median 2p-RSE, which correlates closely with ROC curve data (a well established method of assessing quality), but unlike ROC curves, can be assessed in all samples, not just those with known CN changes. By comparing 2p-RSE results in matched fresh frozen and FFPE samples, we find that CN performance is only modestly reduced in the FFPE samples.

One of the challenges in studying FFPE samples is the variety of processing techniques used by different institutions over time, which may significantly impact sample and thus data quality. We tested MIP CN quantitation in samples sets that originated from seven different sites including five in the US, and one each in the UK and China (Table 1). They represented many different tissue types (bladder, breast, colorectal, kidney, liver, and liver metastases of colorectal tumors), collection methods, storage times (blocks ranged in age from five months to over 20 years) and processing methods (Dana Farber samples were H&E stained with subsequent microdissection).

Only a handful of previous studies have looked at CN in matched FFPE and frozen samples (and generally in less than 20 samples) and while some see much worse performance in FFPE samples, others find the same general patterns of amplifications and gains in matched samples and hence claim similar performance in FFPE and frozen samples [5-7,9,10,27]. But finding the same general pattern does not prove equal performance in FFPE and frozen tissues. We believe that genotype results suggest that other allelic CN technologies may have substantially worse performance in FFPE than frozen samples.

For any CN technology based on a genotyping platform (essentially all allelic CN technologies), one might expect genotyping performance to correlate with CN performance. Consistent with this, in our study CN performance correlates with genotype call rate (r^2 ^= 0.54). Using genotype call rate as a surrogate metric for CN quality, we find only a slight performance decrease in FFPE versus fresh frozen tumors (98.7% vs. 97.5%). In contrast, other studies see substantial drops in call rates (from 93–95% in fresh frozen to 75–91% in FFPE) [5,6,9,27]. (The extremely high genotype concordance we see for samples from the same individual rules out the possibility that our call rates are artificially inflated by adding poor markers.)

The extremely high concordance we find between normal fresh frozen and FFPE samples (99.99%) and tumor fresh frozen and FFPE samples (99.90%) also argues that FFPE samples have little extra variability in our assay. Other studies measure much lower concordance between tumor fresh frozen and FFPE samples (92–98%) and one study comparing normal fresh frozen and FFPE samples also found lower concordance (99.4%) [6,8,9,27]. Finally, the low rate of incompatible genotypes we find between normal and tumor samples (0.01% for fresh frozen, 0.1% for FFPE) suggest that MIP performance is only slightly worse in FFPE samples than fresh frozen. We found no data on genotype incompatibility in other studies.

It should be possible to use the 2p-RSE (or a similar metric) to compare samples within each of these previous studies to determine the relative performance of FFPE and frozen samples within each study. We note that the 2p-RSE is not suited to comparing CN data generated using different platforms, protocols, or algorithms (i.e. across these studies). For example, by changing the protocol to saturate all the features, or altering the algorithm to make all calls very close to CN = 2, the 2p-RSE can be greatly artificially reduced but the data has clearly not been improved.

The 2p-RSE can also be to used guide subsequent smoothing of CN data. While this manuscript focuses on unsmoothed use of MIP CN data, smoothing of data over adjacent markers can improve false positive (FP) and negative (FN) rates at the cost of reduced resolution along the genome. The 2p-RSE may be used to determine the degree of smoothing required to obtain specific FP and FN rates.

The MIP platform generates allele-specific CN which provide several advantages over total CN. First, genotypes can be determined and used for sample tracking and data quality assessment. Second, the allele ratio can be helpful in assessing levels of stromal contamination and derivation of true CN in the malignant cells of a tumor (copy number in the tumor is a joint assessment of malignant cells and contaminating normal stromal cells) [28,29]. The allele ratio can also detect copy-neutral LOH events and assess if amplifications involve one or both alleles. Finally, in a collection of samples, it may be possible to detect an allele bias in amplifications or deletions [30]. For example, if a tumor suppressor gene had two common alleles in a population and one of these alleles showed reduced activity, tumors from heterozygous individuals may have preferentially lost the more active allele.

## Conclusion

We have shown that we can obtain high quality copy number and genotype data using the MIP technology in FFPE samples. The ability to obtain high quality allele CN data from limited amounts of degraded FFPE-derived DNA should greatly facilitate the discovery of genomic aberrations as potential diagnostic, prognostic and predictive biomarkers and may point to novel drug targets.

## Competing interests

YW, VC, GKN, RS, MM, and MF are employed by Affymetrix which holds patents for the MIP technology.

## Authors' contributions

YW contributed to study design, protocol development and validation, and performed experiments; MF contributed to study design and writing the manuscript; VC contributed to algorithm development, analysis and writing the manuscript; MM contributed to algorithm development; GKN, RS, PS, JG contributed to protocol development and validation; LZ, ZW, AR, RW, AW, AS, RK, MT, PT, GM contributed to sample acquisition; MB was involved in providing data and the protocol development and validation.

## Pre-publication history

The pre-publication history for this paper can be accessed here:



## Supplementary Material

Additional file 1 **Probes in assay panel.** Click here for file

Additional file 2 **Sample details.** Click here for file

Additional file 3 **Converting between true and apparent CN and AR given y% contamination with normal DNA and an approximately diploid tumor.** Click here for file

Additional file 4 **Complete Copy Number data.** Click here for file

Additional file 5 **Complete Allele Ratio data.** Click here for file
